# Targeted over-expression of endothelin-1 in astrocytes leads to more severe brain damage and vasospasm after subarachnoid hemorrhage

**DOI:** 10.1186/1471-2202-14-131

**Published:** 2013-10-25

**Authors:** Patrick KK Yeung, Jiangang Shen, Stephen SM Chung, Sookja K Chung

**Affiliations:** 1 Department of Anatomy, Li Ka Shing Faculty of Medicine, The University of Hong Kong, Hong Kong SAR, China; 2 School of Chinese Medicine, The University of Hong Kong, Hong Kong, SAR, China; 3 Research Center of Heart, Brain, Hormone and Healthy Aging, The University of Hong Kong, Hong Kong, SAR, China; 4 Division of Science and Technology, United International College, Zhuhai, Guandong, China; 5 Department of Anatomy, The University of Hong Kong, 1/F, Laboratory Block, Faculty of Medicine Building, 21 Sassoon Road, Hong Kong, SAR, China

**Keywords:** Subarachnoid hemorrhage, Vasospasm, Endothelium, Astrocytes, Brain edema

## Abstract

**Background:**

Endothelin-1 (ET-1) is a potent vasoconstrictor, and astrocytic ET-1 is reported to play a role in the pathogenesis of cerebral ischemic injury and cytotoxic edema. However, it is still unknown whether astrocytic ET-1 also contributes to vasogenic edema and vasospasm during subarachnoid hemorrhage (SAH). In the present study, transgenic mice with astrocytic endothelin-1 over-expression (GET-1 mice) were used to investigate the pathophysiological role of ET-1 in SAH pathogenesis.

**Results:**

The GET-1 mice experienced a higher mortality rate and significantly more severe neurological deficits, blood–brain barrier breakdown and vasogenic edema compared to the non-transgenic (Ntg) mice following SAH. Oral administration of vasopressin V_1a_ receptor antagonist, SR 49059, significantly reduced the cerebral water content in the GET-1 mice. Furthermore, the GET-1 mice showed significantly more pronounced middle cerebral arterial (MCA) constriction after SAH. Immunocytochemical analysis showed that the calcium-activated potassium channels and the phospho-eNOS were significantly downregulated, whereas PKC-α expression was significantly upregulated in the MCA of the GET-1 mice when compared to Ntg mice after SAH. Administration of ABT-627 (ET_A_ receptor antagonist) significantly down-regulated PKC-α expression in the MCA of the GET-1 mice following SAH.

**Conclusions:**

The present study suggests that astrocytic ET-1 involves in SAH-induced cerebral injury, edema and vasospasm, through ET_A_ receptor and PKC-mediated potassium channel dysfunction. Administration of ABT-627 (ET_A_ receptor antagonist) and SR 49059 (vasopressin V_1a_ receptor antagonist) resulted in amelioration of edema and vasospasm in mice following SAH. These data provide a strong rationale to investigate SR 49059 and ABT-627 as therapeutic drugs for the treatment of SAH patients.

## Background

Subarachnoid hemorrhage (SAH) is a subset of stroke, which occurs due to bleeding between the brain and the meninges in the subarachnoid space. Patients with SAH suffer from fatal complications, such as rapid development of cerebral vasogenic brain edema, and rise of intracranial pressure, mainly due to hematoma [1]. One third of patients with SAH also suffer from secondary ischemia due to the delayed narrowing of intracranial arteries (vasospasm). Vasospasm results in the interruption of blood flow to the vital parts of the brain causing morbidity and mortality in up to 30% patients [2]. Vasopressin receptor (V_1_ or V_2_) antagonists have been evaluated in numerous studies using different brain injury paradigms to investigate their effects on attenuating edema development after brain injury in animal models [3-5]. Until now, the pathophysiological aspects and mechanisms of SAH-induced vasospasm have not been completely elucidated, and the treatment for SAH-induced vasospasm remains one of the major challenges in neurosurgery.

Break down of red blood cells and release of oxyhemoglobin is the putative cause of delayed vasospasm after SAH [6-9]. One of the oxyhemoglobin-mediated cerebral vasospasm mechanisms is the release of a potent vasoconstricting agent, endothelin-1 (ET-1) [10,11]. ET-1 is a potent vasoconstrictor originally isolated from aortic endothelial cells. However, ET-1 has also been detected in several other types of brain cells, including endothelial cells, neurons and astrocytes [12-14]. It has been demonstrated that activated mononuclear leukocytes are involved in ET-1 production under SAH [15]. Additionally, the release of ET-1 can be stimulated by oxyhemogloblin or thrombin in endothelial and smooth muscle cells [16]. However, it is still unclear whether astrocytic ET-1 and the mechanisms of its release are responsible for cerebral vasospasm development. Several clinical studies have established a correlation between elevated ET-1 levels in plasma and cerebral spinal fluid, and cerebral vasospasm-mediated ischemic damage after SAH [17-19], indicating that ET-1 may also be produced during delayed ischemia after SAH.

Astrocytic ET-1 has been shown to play a role in the pathogenesis of cerebral ischemic injury. Previously, we have demonstrated that transgenic mice (GET-1 mice) that over-express endothelin-1 (ET-1) specifically in the astrocytes are more susceptible to brain damage, including increased infarct volume, hemispheric swelling as well as cerebral water content, upon transient focal ischemia induced by middle cerebral artery occlusion (MCAO). We have also demonstrated that GET-1 mice develop more severe cytotoxic edema when induced by water intoxication [20,21]. However, it is still unclear whether astrocytic ET-1 also plays an important role in vasogenic edema formation.

In the present study, transgenic mice with astrocytic endothelin-1 (GET-1 mice) over-expression were used to investigate the pathophysiological role(s) of ET-1 in SAH pathogenesis with the aim of dissecting the mechanisms involved in the formation of secondary ischemia in SAH. A better understanding of the molecular mechanisms involved in SAH formation may help in formulation of therapeutic strategies for the treatment and management of the disease.

## Methods

### Mouse subarachnoid hemorrhage model

Both Ntg and GET-1 mice were housed under controlled diurnal lighting conditions and allowed free access to food and water. The protocol of this study was reviewed and approved by the Committee on the Use of Live Animals in Teaching and Research in the University of Hong Kong.

SAH was induced in mice by the artery puncture method as previously described [22]. In brief, age-matched Ntg or GET-1 mice were anesthetized with gas (2% halothane in 70% N_2_O/30% O_2_ for induction and 1% halothane in 70% N_2_O/30% O_2_ for maintenance) [23]. Regional cerebral blood flow (rCBF) was monitored and recorded with a Laser-Doppler system (PeriFlux 5001 and Perisoft software) during the whole surgical procedure to confirm successful SAH induction. The average value of rCBF was recorded one minute before induction of SAH served as the control value. SAH was induced, and rCBF was recorded for 20 minutes after SAH induction. The rectal temperature was maintained at 37 ± 0.5°C with a temperature control system (FHC, Brunswick, ME, USA). The right carotid artery was identified along with all its extracranial branches, and the external carotid artery was dissected. A sharpened 5–0 monofilament suture was advanced into the external carotid artery past the common carotid bifurcation and into the internal carotid artery. The suture was advanced distally into the intracranial internal carotid artery until resistance was felt and then pushed 3 mm further through the right anterior cerebral artery (ACA) near its intracranial bifurcation. The suture was then withdrawn into the external carotid artery immediately, which allowed reperfusion of the right internal carotid artery (ICA) leading to SAH. After the surgery, the mice were returned to the Intensive Care Unit (ICU) to facilitate recovery from anesthesia.

### Neurological evaluation following SAH

A subset of animals was used for neurological evaluation in the SAH experiment. A 100-point neurological scoring system (scoring scale 0 to 100), which assessed the general behavioral deficit, cranial nerve reflexes deficit, motor deficit, sensory deficit and coordination deficit, was used to evaluate the neurological deficit of mice 24 hours after SAH and continued for three days [24]. A general behavioral score (0–40) was derived from spontaneous activity and respirations. A cranial nerve reflex score (0–20) was derived from examination of olfactory, vision, blinding reflex, whisker movement and hearing response to stimulus. A motor score (0–10) was derived from examination of symmetry of limb movement. A sensory score (0–10) was derived from the response of mice while their tails were being pinched. A coordination score (0–20) was derived from examination of traveling ledge balance, righting reflex, front paws reaching and retreatment response. Mice were sacrificed afterwards and brain tissues were collected for further analysis. The behavioral tests were performed blinded to the genotypes.

### Perfusion-fixation and vascular diameter measurement

At 72 hours after SAH induction, when vasospasm has been reported to peak in mice models [25], cerebral vascular perfusion and vascular diameter measurement were performed as described previously [26]. After neurological deficit evaluation, mice were anesthetized. The chest was opened, and the aorta was cannulated with a blunted 20-gauge needle. Flexible plastic tubing connected to the needle was used to deliver infusion solutions by manual pulsatile syringe pressure, and the tubing was connected to a 30-ml syringe. An incision was made in the right atrium to allow outflow of perfusion solutions. 20 ml of 0.9% NaCl was infused followed by 10% formalin and gelatin-india ink solution. The dead mice were refrigerated for 24 hours to allow gelatin solidification, and thereafter the brains were harvested and stored in 4% neutral buffered formaldehyde. The cerebral vasculature was photographed by using a video-linked microscope (Fluorescence Stereo Microscope, MZ FLIII, Leica). At 72 hours after SAH induction, the diameter of the left and right MCA was measured at the site 1 mm distal to the MCA-ACA bifurcation [27]. All procedures were performed blinded to the genotypes.

### Water content measurement

Different groups of animals were used for the water content measurement. Brain tissue was removed for the brain water content analysis at 24 hours after SAH. The brain was weighted immediately to obtain the wet weight. The brain was then dried in an oven at 105°C for 48 hours and weighed again to obtain the dry weight. The percentage of water content was calculated as [(wet weight-dry weight)/wet weight] × 100% [28].

### Evans Blue extravasation

Different groups of animals were used for the Evans Blue (EB) extravasation. On Day 3 (72 hours) after SAH induction, 0.1 ml of 4% EB in saline was injected intravenously into the mice and allowed to circulate for 3 hours [29]. For extraction of EB from brain, the brain was placed in 1 ml PBS and homogenized by sonication. The homogenate was centrifuged at 15,000 rpm for 30 mins and 0.5 ml supernatant was added to an equal volume of trichloroacetic acid. The mixture was incubated at 4°C overnight and centrifuged at 15,000 g at 4°C for 30 mins after incubation. The supernatant was collected, and EB was measured at an excitation wavelength of 620 nm and an emission wavelength of 680 nm using a fluorescence spectrophotometer. EB concentrations were calculated using the standard curve, and expressed as μg/g brain tissue [30].

### Immunocytochemical (ICC) analysis

On Day 3 after SAH induction, brain samples were collected after neurological evaluation, and fixed with 4% paraformaldehyde. MCA was isolated according to a published protocol [31]. In brief, the first cut was made at the groove between the forebrain and cerebellum (−4.5 mm from bregma), then the second cut at −6.5 mm from bregma, and the final cut at 3 mm anterior to the groove (−1.5 mm from bregma). The anterior portion of the forebrain was then turned 90° and two sagittal cuts were made at the midline crossing the olfactory tract to expose the cross section of the MCA [31]. 7 μm thick coronal brain slices were used for the ICC study. Brain sections were incubated with antibodies against GFAP (1:2000, Z0334, DAKO, Carpinteria, CA, USA), nNOS (1:200, 610310, BD Transduction Lab., USA), eNOS (1:200, 610298, BD Transduction Lab., USA), *p*-eNOS (1:100, 9571, Cell Signaling Technology, USA), ET_A_R (1:100, 324758, Calbichem, Merck, Germany), Maxi K^+^α (1:500, 444910, Cabiochem, Merck, Germany ), PKC-α (1:500, abcam ab4124, UK) and endothelin 1 (1:250, abcam ab2786, UK). Signals were visualized by Vectastain ABC kit (Vector Laboratories, Burlingame, CA, USA) with 3,3’-diaminobenzidine tetrahydrochloride (Zymed, South San Francisco, CA, USA). All the conditions were followed as described in our previous study [20].

### Quantification of ICC photomicrographs

Pictures of the brain sections with positive staining (GFAP: magnified 50×; MCA: magnified 100×) were analyzed with the software ImageJ [32]. For analyzing the staining of the MCA, the entire vascular wall of the MCA with staining was marked and the intensity of the staining was measured [33]. The value was expressed as mean ± SEM. All immunocytochemical photomicrograph quantifications were performed in a blinded manner and the results were expressed relative to Ntg sham group, which were arbitrarily assigned a value of 100%.

### Western blot analysis

Proteins were extracted from the brains of sham and SAH groups of Ntg and GET-1 mice after 3 days. The brain tissues were homogenized in ice-cold lysis buffer (50 mM Tris–HCl, pH 8.8, 150 mM NaCl, 5 mM EDTA, 0.5% sodium deoxycholate, 0.5% NP-40, plus proteinase inhibitor cocktail). Homogenate was centrifuged at 4°C, 3000 g for 5 mins, and the supernatant was used for the Western blot analysis. Blots were incubated with antibodies against PKC-α (1:500, ab4124, abcam, UK), phospho-PKC-α (1:1000, Cat. no. #9375, Cell Signaling) and α-tubulin (1:5000, sc-5286, Santa Cruz). Signals were visualized by ECL (Amersham) and quantitated using PhotoImager (Molecular Dynamics). Values for protein levels were given as relative percentage to Ntg sham group after normalization with individual α-tubulin levels for equal loading.

### Drug treatments

For the drug treatment, ET_A_ receptor antagonist, ABT-627, was dissolved in 0.25 M NaCO_3_. Both Ntg and GET-1 mice received an intraperitoneal injection of 10 mg/kg ET_A_ receptor antagonist, ABT-627 (a generous gift from Dr. Ruth Wu-Wong), 5 mins after onset of SAH. For the vasopressin V_1a_ receptor antagonist treatment, SR 49059 was dissolved in 10% DMSO. The mice received an intraperitoneal injection of 30 mg/kg SR 49059, 5 mins after SAH. The dosage concentration used was similar to the effective dosage concentration used in a previous study [34]. For all the drug tests, vehicle-treated mice were used as controls.

## Results

### GET-1 mice had a higher death rate than the Ntg mice after SAH

**Figure 1:**
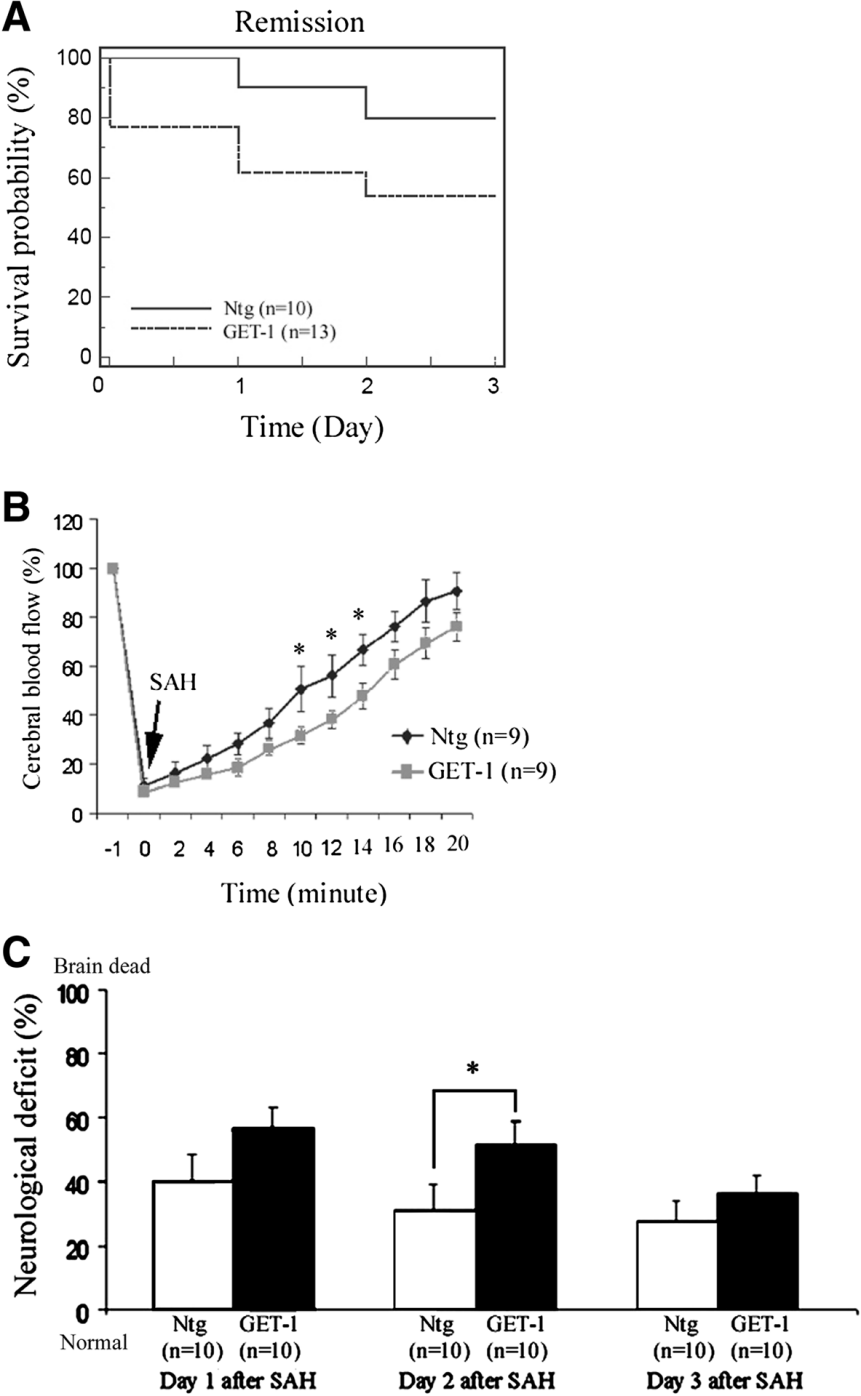
**Effects of subarachnoid hemorrhage induction in Ntg and GET-1 mice after subarachnoid hemorrhage. (A)** Kaplan-Meier curves show the survival data of the Ntg (solid line, n = 10) and the GET-1 (broken line, n = 13) mice after subarachnoid hemorrhage. **(B)** Line graph shows the cerebral blood flow of Ntg and GET-1 mice during SAH. **(C)** Histogram comparing the neurological deficit scores for the Ntg (white bar) and GET-1 (black bar) mice after SAH for 3 days. (*P < 0.05, Mann-Whitey test; n = 10 for Ntg and GET-1 mice).

The survival rate of Ntg, and GET-1 mice was 100% and 77%, respectively, 24 hours after SAH. The survival rate of Ntg mice decreased steadily on Day 2 (90%) and Day 3 (80%) after SAH. However, GET-1 mice showed a substantial drop in the survival rate, 61% and 54% on Day 2 and Day 3, respectively. GET-1 mice were more susceptible to SAH damage with a death rate of about 50% three days after SAH (Figure 1A).

### Laser-doppler flowmetry

Upon subarachnoid hemorrhage induction, there was a dramatic and immediate decrease in cerebral perfusion (to < 20% of baseline) in both Ntg and GET-1 mice (Figure 1 B). The relative cerebral blood flow (rCBF) was recovered gradually to about >80% in the Ntg mice and about 70% in the GET-1 mice after 20 minutes. Ntg mice showed significantly higher rCBF when compared with the GET-1 mice during the reperfusion time points of 10, 12 and 14 minutes (Ntg: 50.6 ± 8.4, 55.9 ± 6.5, 66.5 ± 6.3 vs GET-1: 26.9 ± 3.8, 34.1 ± 4.8, 43.6 ± 6.1, n = 9, *P < 0.05 by Mann–Whitney test). In the sham groups, no changes of rCBF were observed in both Ntg and GET-1 mice (~ 100%) and at all time points, the rCBF of sham groups was significantly higher than the SAH-induced group (data not shown).

### GET-1 mice exhibited more severe neurological dysfunction than the Ntg mice after SAH

The neurological deficits of the Ntg and GET-1 mice were evaluated for 3 days after SAH. As indicated in the neurological deficit score, the Ntg mice exhibited the greatest neurological dysfunction on Day 1 after SAH (40.0± 7.5; n = 10) and recovered gradually on Day 2 (31.1± 7.6; n = 10) and Day 3 (23.1± 6.4; n = 10). In the GET-1 mice, the neurological dysfunction on Day 1 after SAH was 56.8± 8.1(n = 10) and the mice gradually recovered on Day 2 (51.7.1± 7.0; n = 10) and Day 3 (39.2± 5.3; n = 10). During the whole period of neurological deficit assessment, GET-1 mice showed higher dysfunction compared to Ntg mice after SAH, with a significant difference on Day 2 (*P < 0.05 by Mann–Whitney test) (Figure 1C).

### GET-1 mice were more susceptible to blood-brain barrier (BBB) breakdown than Ntg mice after SAH

**Figure 2:**
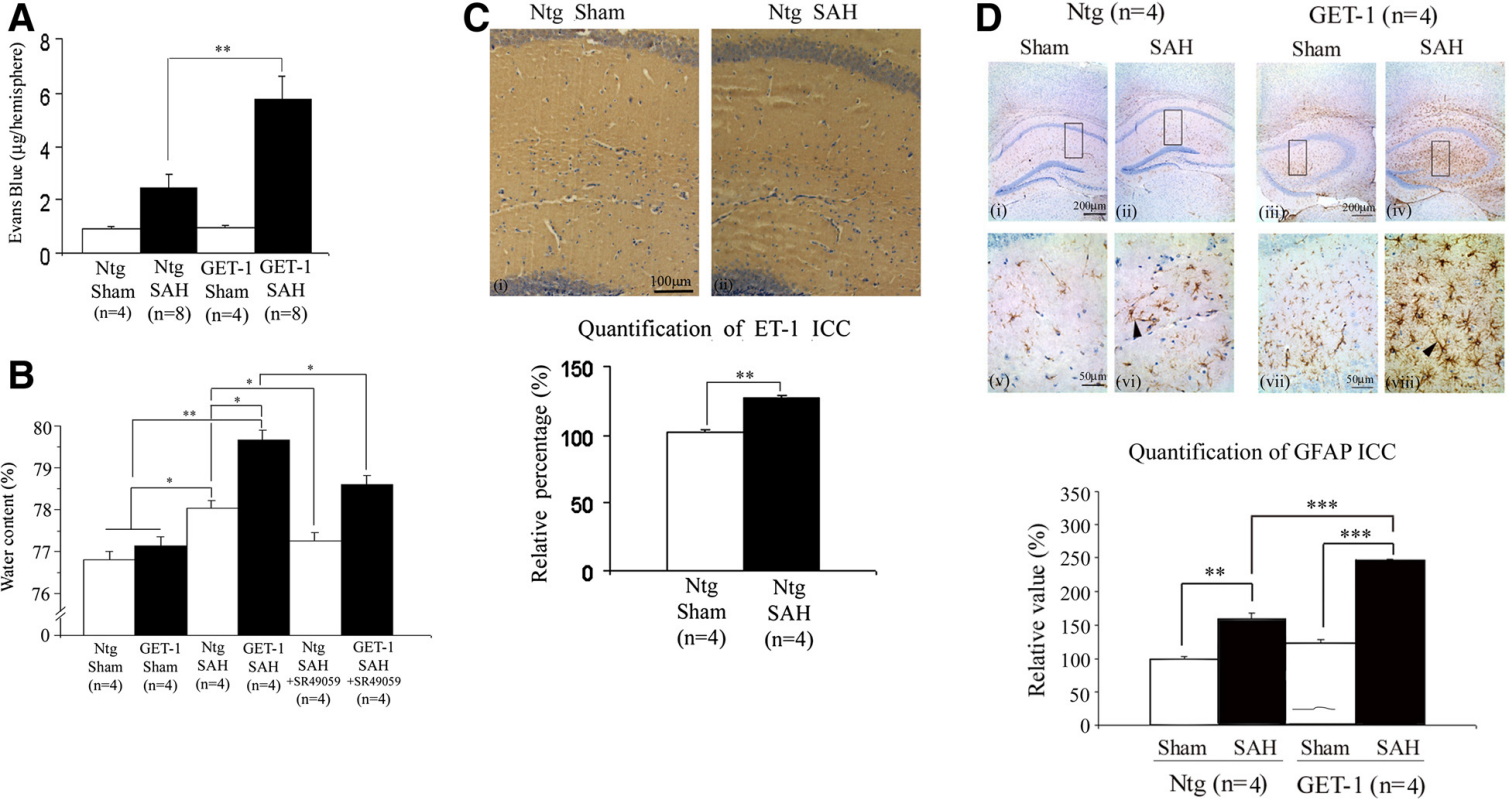
**GET-1 mice showed more severe brain edema with upregulation of ET-1 and GFAP expressions after subarachnoid hemorrhage. (A)** Analysis the integrity of the BBB with the EB extravasation in Ntg and GET-1 brain after SAH. (*P < 0.05, ANOVA followed by Bonferroni’s test; n = 4 for the sham groups and n = 8 for the SAH groups). **(B)** Histogram comparing the water content of the Ntg and GET-1 mice with or without SR 49059 treatment after SAH (*P < 0.05, ANOVA followed by Bonferroni’s test; n = 4 for each group of mice). **(C)** Immunocytochemical analysis of ET-1 expression in Ntg brain after SAH. Representative micrograph shows the expression of ET-1 in the hippocampal region of the Ntg brain after Sham (i) and SAH (ii) (n = 4 for each group). Histogram below shows the quantification results (relative value in percentage) of ET-1 Sham and SAH of Ntg brain sections using software ImageJ. (**P < 0.01, student t-test; n = 4 for each group of mice). **(D)** Immunocytochemical analysis of GFAP expression after SAH. Representative micrograph shows the localization and expression of GFAP in the astrocytes of the hippocampus of the Ntg and GET-1 brain at (i-iv) low and (v-viii) high magnification (n = 4 for each group). The GFAP signals in the astrocytes at the SAH groups of the Ntg and GET-1 brains is increased, and they have longer processes (arrowheads). Histogram below shows the quantification results (relative value in percentage) of GFAP Sham and SAH of Ntg and GET-1 brain sections using software ImageJ. (*P < 0.05, **P < 0.01, ***P < 0.005, ANOVA followed by Bonferroni’s test; n = 4 for each group of mice).

To further understand the role of astrocytic ET-1 in SAH-induced neurological dysfunction in GET-1 mice, the BBB integrity in both Ntg and GET-1 mice was investigated with Evans Blue (EB) extravasation experiment (Figure 2A). At 24 hours after SAH, the amount of EB leakage was increased in NTg and GET-1 mice when compared with their sham groups. Most importantly, a significant elevation of EB leakage was observed in GET-1 brains (Ntg: 2.42 ± 0.54 μg/hemisphere vs GET-1: 5.74 ± 0.87 μg/hemisphere, n = 8, **P < 0.01 by Mann–Whitney test) (Figure 2A), indicating increased BBB permeability and more BBB breakdown in the brains of GET-1 mice after SAH.

### GET-1 mice accumulated more water in their brains than the Ntg mice after SAH, and SR 49059 significantly reduced the water content in the GET-1 mice after SAH

GET-1 mice brains showed more severe BBB breakdown, suggesting that those mice experienced more severe vasogenic edema after SAH. A significant difference was observed in the cerebral water content between the Ntg and the GET-1 mice (Ntg: 78.0 ± 0.20%; GET-1: 79.60 ± 0.23%; *P < 0.05 by Mann–Whitney test) suggesting that the GET-1 mice were more susceptible to SAH-induced cerebral edema (Figure 2B).

A previous study reported that arginine vasopressin (AVP) V_2_ receptor antagonist ameliorates cytotoxic edema and SAH-induced cerebral edema formation [35]. To investigate whether another subtype of vasopressin receptor is also involved in SAH-induced vasogenic edema development, V_1a_ receptor antagonist, SR 49059, was tested. A significant reduction of cerebral water content was observed in both Ntg and GET-1 mice treated with SR 49059 when compared with their vehicle-treated group (Ntg vehicle-treated: 78.03 ± 0.18%; Ntg SR 49059-treated: 77.24 ± 0.21%, GET-1 vehicle-treated: 79.68 ± 0.20%; GET-1 SR 49059-treated: 78.60 ± 0.21%, *P < 0.05 by Mann–Whitney test) (Figure 2B). For both genotypes, all mice survived 24 hours after SR 49059 treatment.

### Upregulation of endothelin-1 (ET-1) in the wild-type mice brain after SAH

To investigate whether ET-1 is involved in the pathophysiology of SAH, the expression level of ET-1 was examined by the immunocytochemical analysis. After SAH, the Ntg brains showed a significant upregulation of ET-1 expression in the hippocampal region, where astrocytes are highly re-activated under stress conditions, when compared with the sham control. This suggested that astrocytic ET-1 played a role in the pathophysiology of SAH condition (Figure 2C).

### Increased glial fibrillary acidic protein (GFAP) in the GET-1 mice brain after SAH

To further understand the role of over-expressing astrocytic ET-1 in the pathogenesis after SAH, immunocytochemistry was used to determine the expression and cellular location of GFAP.

GFAP is an intermediate filament cytoskeletal protein expressed primarily by astroglia. Increased expression of GFAP represents astroglial activation and gliosis in many neurodegenerative diseases; therefore, GFAP has been used as an indicator of brain injury [36-38]. After SAH, both Ntg and GET-1 brains showed a significant increase in the GFAP expression in the hippocampal region when compared with their sham control. At a higher magnification, GFAP-labeled astrocytes were stained brownish and appeared star-shaped. The GFAP staining in the brain of the GET-1 mice was significantly more intense and widespread in the hippocampal region compared to the Ntg mice. Furthermore, longer astrocytic processes were observed in the GET-1 mice brains (Figure 2D).

### GET-1 mice exhibited a more severe vasospasm after SAH

**Figure 3:**
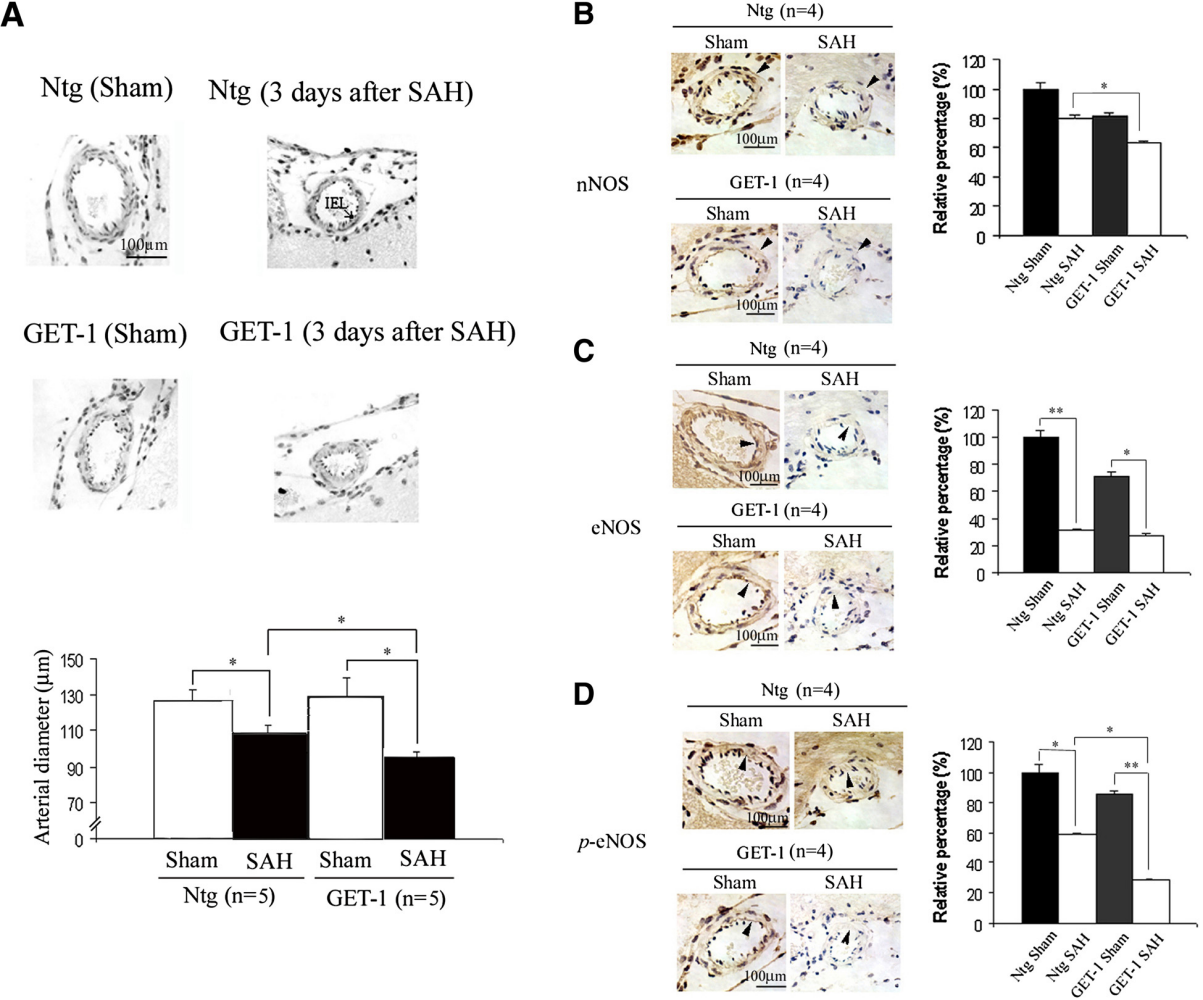
**Vasospasm analysis and NOS expressions in MCA after subarachnoid hemorrhage. (A)** Representative pictures show the histological analysis of MCA. Vasospasm is observed in both Ntg and GET-1 brain after SAH when compared to that of their sham group (n = 5). Histogram shows the arterial diameter measurement of MCA in Ntg and GET-1 after SAH. (*P < 0.05, Mann-Whitey test; n = 5 for Ntg and GET-1 mice). Expression of NOS in MCA after SAH. Representative photos shows the **(B)** nNOS, **(C)** eNOS and **(D)***p*-eNOS expressions of sham and SAH of MCA in Ntg and GET-1 brain. Arrowheads show the expression of nNOS (tunica adventitia) and eNOS/*p*-eNOS (tunica intima), (n = 4 for each group of mice). Histograms show the quantification results (relative value in percentage) of immunocytochemistry of sham and SAH of Ntg and GET-1 MCA sections. (*P < 0.05, **P < 0.01, ANOVA followed by Bonferroni’s test; n = 4 for each group of mice).

To investigate the role of astrocytic ET-1 on SAH-induced vasospasm, MCAs were isolated and examined from both genotypes after SAH. Vasospasm in the MCA was observed in both Ntg and GET-1 when compared to their sham groups, and the MCA in the brains of the GET-1 mice showed more severe constrictions compared to the Ntg mice (Sham: Ntg 126.2 ± 7.4 μm vs GET-1 132.2 ± 10.4 μm; SAH: Ntg 106.5 ± 2.3 μm vs GET-1 99.7 ± 3.1 μm, n = 5, *P < 0.05 by Mann–Whitney test) (Figure 3A).

### Immunocytochemical studies of NOS expression in MCA

Nitric oxide (NO), also known as an Endothelium-Derived Relaxing Factor, which is generated by vascular endothelium and is one of the important factors that regulates smooth muscle tone [39]. nNOS is produced in the adventitia and eNOS is produced in intima [40], and studies have shown that both neuronal (nNOS) and endothelial nitric oxide synthase (eNOS) depletion underlies vasospasm [39,41]. ICC was performed to investigate the expression of NOs in GET-1 mice brains after SAH. After SAH, the expression of nNOS in the layer of adventitia (arrows) of MCA was reduced in both genotypes when compared to their sham groups. Significant reduction in nNOS was observed in the MCA of GET-1 mice compared to the Ntg mice after SAH (Figure 3B). Furthermore, both eNOS and *p*-eNOS expressions were significantly reduced in both of Ntg and GET-1 MCA after SAH compared to their sham groups. Additionally, *p*-eNOS expression of GET-1 MCA after SAH was significantly reduced when compared to that of Ntg MCA (Figure 3C and D).

### GET-1 mice showed an upregulation of PKC-α/phospho-PKC-α expression in the brain after SAH

**Figure 4:**
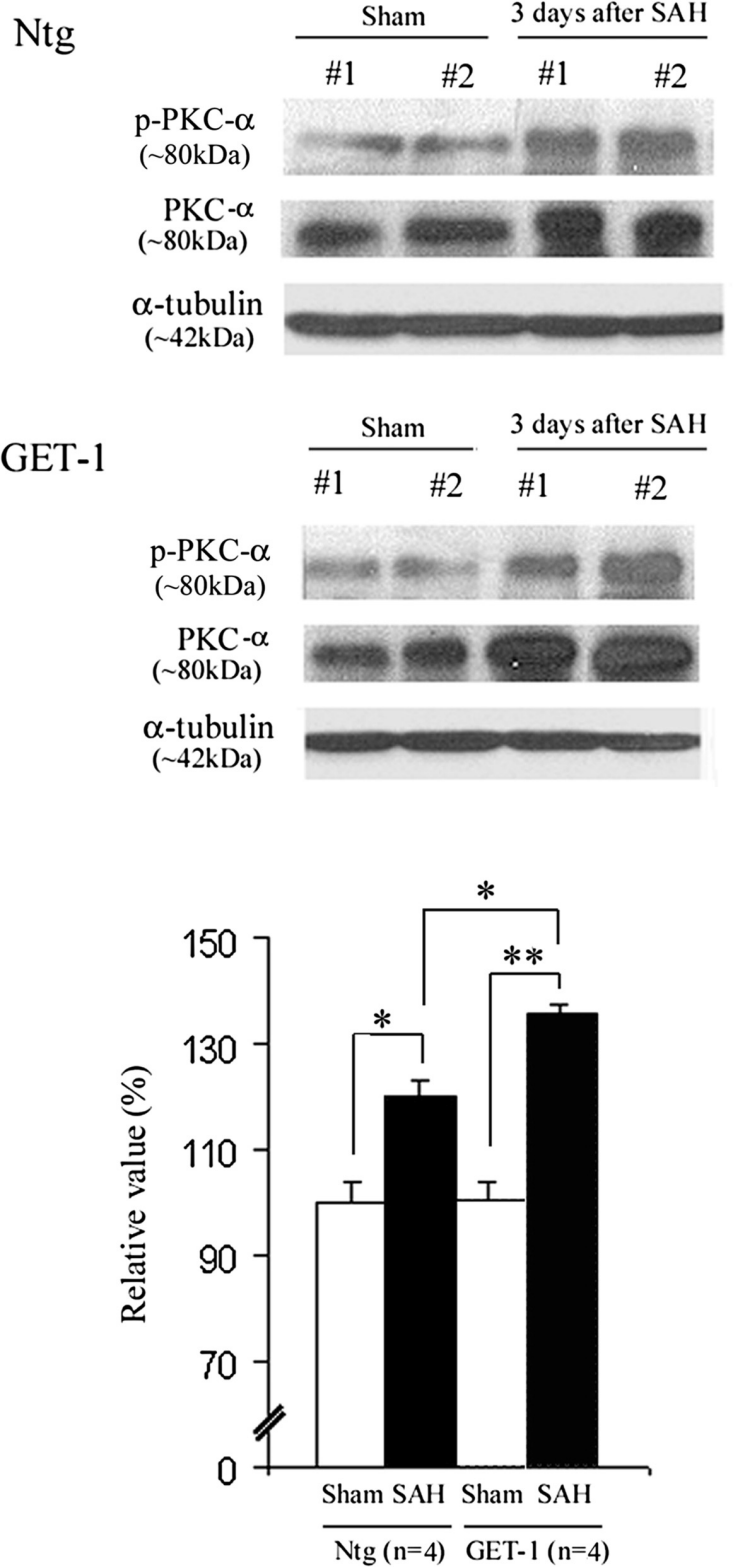
**Western blot analysis of PKC-α and p-PKC-α in Ntg and GET-1 brain after SAH.** Representative blots show PKC-α and p-PKC-α expression in the sham and SAH groups of Ntg and GET-1 brain. Histogram below shows the quantification (relative percentage) of the Western blot. (*P < 0.05, **P < 0.01, Mann-Whitey test; n = 4 for Ntg and GET-1 mice).

In SAH, ET-1 induces the development of vasospasm through enhancement of PKC-α activity, and the increased-PKC-α activity inhibits eNOS production [42,43]. To investigate whether PKC-α mediates astrocytic ET-1 induced downregulation of NOS, Western blot analysis was performed. Significant upregulation of PKC-α and *p*-PKC-α was observed in the brain of both Ntg and GET-1 mice after SAH when compared to their sham groups (Ntg Sham: 100 ± 3.7% vs Ntg SAH: 119.7 ± 4.6%, P < 0.05; GET-1 Sham: 100.5 ± 3.7% vs GET-1 SAH: 135.8 ± 1.6% P < 0.01, Mann–Whitney test; n = 4). Significant difference was observed between Ntg and GET-1 mice brain after SAH (Ntg SAH; 119.7 ± 4.6% vs GET-1 SAH 135.8 ± 1.6% P < 0.05, Mann–Whitney test; n = 4) (Figure 4).

### GET-1 MCA showed downregulation of potassium channels after SAH

**Figure 5:**
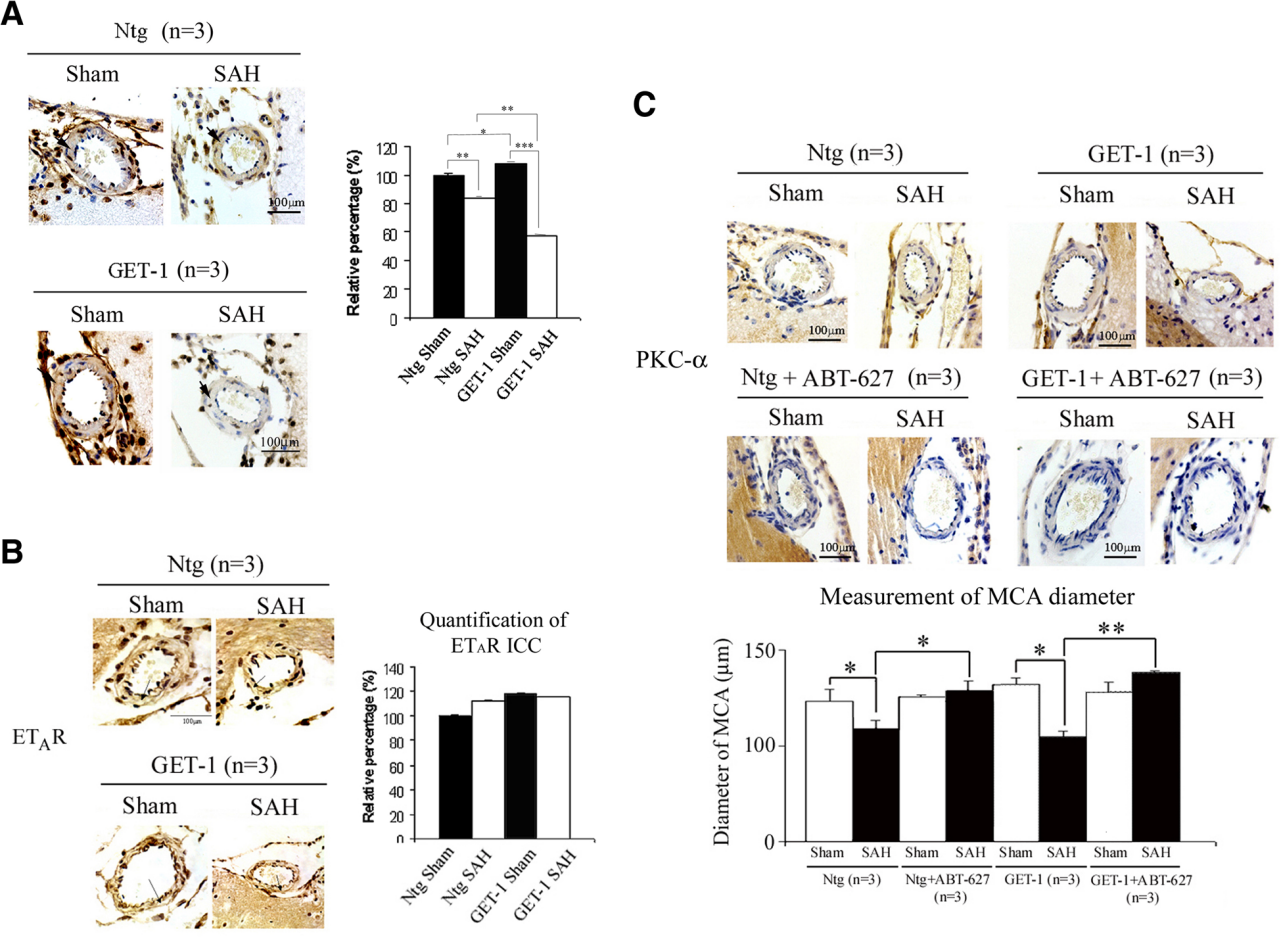
**Immunocytochemical analysis of Ca**^**2+**^**-activated K**^**+ **^**channel and ET**_**A**_**R expressions in Ntg and GET-1 MCA after SAH. (A)** Representative micrograph shows the localization and expression of Ca^2+^-activated K^+^ channel in the MCA of the Ntg and GET-1 after SAH. (n = 3 from each group of mice). Histogram shows the quantification results (relative value in percentage) of immunocytochemistry of sham and SAH of Ntg and GET-1 MCA sections. (*P < 0.05, **P < 0.01, ***P < 0.005, ANOVA followed by Bonferroni’s test; n = 3 for each group of mice). **(B)** Representative micrograph shows the localization and expression of ET_A_R in the smooth muscle cells (arrows) of MCA in Ntg and GET-1 mice. Histogram shows the quantification results (relative value in percentage) of immunocytochemistry of sham and SAH of Ntg and GET-1 MCA sections. (*P < 0.05, **P < 0.01, ANOVA followed by Bonferroni’s test; n = 3 for each group of mice). **(C)** Representative micrograph shows the immunocytochemical expressions of PKC-α and the diameters of MCA after treating with ET_A_R antagonist ABT-627. Histogram below shows the measurements of MCA diameter. (*P < 0.05, **P < 0.01, Mann-Whitey test; n = 3 for Ntg and GET-1 mice).

The vascular tone of the smooth muscle is regulated by the K^+^ membrance conductance and studies have shown that reduced expression of the potassium channels might contribute to vasospasm after SAH [44,45]. To investigate the expression of the Ca^2+^-activated K^+^ channel in the MCA after SAH, immunocytochemical analysis was performed. Significant downregulation of Ca^2+^-activated K^+^ channel expression was observed in both Ntg and GET-1 MCA after SAH compared to their sham groups. GET-1 MCA after SAH showed a significant reduction of Ca^2+^-activated K^+^ channel expression compared to the Ntg MCA (Figure 5A), suggesting that astrocytic ET-1 mediated-SAH induces a more severe dysfunction of K^+^ channel that might lead to cerebral vasospasm.

### ET_A_ receptor antagonist ameliorated vasospasm after SAH

ET-1 is one of the important factors in development of vasospasm after SAH and plays a role in vascular tone of smooth muscle contraction through ET_A_ receptor [46]. ET_A_ receptor is localized in the vascular smooth muscle cells and mediates vascular contraction [47]. When compared to their sham groups, the expression of ET_A_ receptor only slightly increased in the smooth muscle cells (tunica media) (arrows) in both Ntg and GET-1 MCA after SAH (Figure 5B). To determine if ET_A_ receptor plays a role in the development of vasospasm, ABT-627, a competitive ET_A_ receptor anatagonist, was administrated 5 mins after onset of SAH. Treatment with ABT-627 resulted in an improvement in neurological deficits and reduced infarct size after MCAO in GET-1 mice. In Figure 5C, down-regulation of PKC-α was observed in both Ntg and GET-1 MCA after ABT-627 treatment when compared to the vehicle controls. In addition, ABT-627 treatment significantly ameliorated vasospasm in the MCA of both GET-1 and Ntg mice compared to their vehicle controls. ABT-627 treatment resulted in a more promising improvement in GET-1 mice when compared to Ntg mice (Ntg SAH: 114.5 ± 4.9 μm vs Ntg SAH + ABT-627: 133.5 ± 5.1 μm; GET-1 SAH: 104.7 ± 3.1 μm vs GET-1 SAH + ABT-627: 138.6 ± 1.0 μm, *P < 0.05, **P < 0.01 Mann–Whitney test; n = 3). Administration of ET_B_ receptor anatagonist, BQ788, however, did not show any effect in either genotypes (data not shown).

## Discussion

Elevation of ET-1 levels in the cerebrospinal fluid after SAH have been reported in SAH patients [18]. ET-1 is known to be a potent endothelium-derived vasoconstricting agent [48]. In the CNS, ET-1 can be produced by astrocytes, neurons and pituitary cells under normal physiological conditions [12,14,49]. ET-1 is also released from endothelial and smooth muscle cells when stimulated by thrombin and oxyhemoglobin [8,50]. However, to date the source and the release mechanisms of ET-1 are still largely unknown. In order to address whether astrocytic ET-1 plays a role in the development of cerebral edema and vasospasm after SAH, transgenic mice over-expressing ET-1 in astrocytes (GET-1) was used to exaggerate the effects of ET-1 during SAH and delayed ischemia after SAH. The GET-1 mice (from 1 week to 20 weeks old) show higher endothelin-1 mRNA and peptide levels in the brain when compared to Ntg brains. In spite of the increased level of astrocytic ET-1, GET-1 mice appear normal under physiological condition. The cerebrovasculature and the mean artery blood pressure is similar in the GET-1 compared to the Ntg mice, suggesting that the GET-1 mice show no significant difference in the cerebral artery under normal physiological condition [20]. Although GET-1 mice have been shown to have higher endothelin-1 mRNA and peptide levels in the brain when compared to Ntg brains [20], their cerebrovasculatures showed no significant difference to that of Ntg brains. We speculate that the insignificant increased level of ET-1 in GET-1 mice may not cause any exacerbating effects on cerebral vasospasm and edema formation; however, these mice will be more susceptible to these pathophysiological processes under stress conditions, such as SAH when astrocytic ET-1 level will be increased substantially.

Vasopressin levels increase in the plasma and cerebrospinal fluid after SAH, suggesting that the antidiuretic hormone plays a critical role in edema development [51]. SR 49059 is considered to be the most potent and selective non-peptide V_1a_ receptor antagonist in both animals and human [52,53]. Recently, SR 49059 was shown to reduce intracerebral hemorrhagic brain injury-induced cerebral edema in mice [54]. In the present study, arterial puncture technique was used to induce subarachnoid hemorrhage, and GET-1 mice developed more severe brain damage and edema after this procedure. SR 49059 was administrated intraperitoneally to the NTg and GET-1 mice to evaluate the effect of vasopressin V_1a_ receptor antagonist in the edema formation after SAH. The results showed that SR 49059 significantly reduced brain water content in both Ntg and GET-1 mice 24 hours after SAH, which is in agreement with a previous study [55] despite using a different type of hemorrhagic stroke model and paradigm. Collectively, these data suggest that SR 49059 is a promising drug in treating hemorrhagic brain edema and provide a strong rationale to investigate the drug in clinical settings.

Although many studies have reported that ET-1 levels are increased in plasma and CSF during ischemic and hemorrhagic stroke [56,57], it is still unclear whether the production and release of ET-1 is the primary response of the brain cells (astrocytes, endothelial cells, neurons) to the stroke, or the secondary response in which activation of the sympathoadrenal system increases the plasma catecholamine and vasopressin levels [58], which stimulates the release of ET-1 from the peripheral organs. In the present study, over-expression of astrocytic ET-1 in GET-1 mice resulted in a more severe brain damage and vasospasm, suggesting that astrocytes may be one of the major sources of ET-1 production and release under pathological conditions. Previous *in vitro* data suggested that the SAH-induced hypoxia-ischemia in astrocytes accounts for the ET-1 release into the subarachnoid space [56]. The present report provides the first documentation for the significance of astrocytic ET-1 in haemorrhagic stroke in an animal model. Our data demonstrate that overexpression of astrocytic ET-1 excerbates several pathophysiological processes after SAH, and this could be a contributing factor to these processes together with the physiological levels of astrocytic ET-1, however, we could not directly conclude that this is the case. Further studies in animals, such as with targeted deletion of astrocytic ET-1, will be required before drawing the conclusion.

In agreement with other studies, we demonstrate that astrocytic ET-1 also induces vasospasm with a concurrent elevation of PKC-α protein expression and activation [43,59,60]. ET-1 regulates the vascular tone of the cerebral blood vessels through its receptor subtypes, ET_A_ and ET_B_. ET_B_ receptors are known to mediate vasodilation upon localization to the endothelial cells of blood vessels. A recent study shows that the expression of ET_B_ receptors is regulated by initial cerebral blood flow through the MEK-ERK1/2 signaling pathway [61]. ET_A_ receptors are mainly found in smooth muscle cells and are involved in vasoconstriction; therefore, they are crucial in cerebral vasospasm [62]. In the present study, immunocytochemical analysis of ET_A_ receptor expression in MCA showed an insignificant change in both Ntg and GET-1 after SAH, which is in agreement with the previous finding that the expression of smooth-muscle ET_A_ receptors and their mRNA level is unchanged or slightly increased in the cerebral arteries after SAH [63,64]. It is demonstrated that an increased coupling of the smooth muscle ET_A_ receptor with the second cascade probably contributes to the development of cerebral vasospasm [64]. ET_A_ receptor antagonists have been used in numerous studies in alleviating SAH-induced cerebral vasospasm [65-67]. However, other studies have also reported that ET_A_ receptor antagonists have the potential adverse effects such as hypotension and pneumonia. Moreover, there are no significant differences in mortality or improving outcomes in the phase 3 clinical trials investigating ET_A_ receptor antagonists as a therapeutic strategy for vasospasm [68-71]. However, ET_A_ receptor antagonists, such as clazosentan, have been used in alleviating SAH-induced cerebral vasospasm [72]. In a clinical study, only high doses of clazosentan resulted in a significantly reduced vasospasm-related morbidity or all-cause mortality within 6 weeks post SAH, but not at longer time points [69], suggesting that ET_A_ receptor antagonist could be used for treating vasospasm. However, the interference by other drugs taken by the patients during the clinical study may reduce the efficiency of the clazosentan at a later time point. In the current study, ET_A_ receptor antagonist ABT-627 effectively attenuated SAH-induced vasospasm in both Ntg and GET-1 mice, and suggested that pathways elicited by astrocytic ET-1 through ET_A_ receptor, but not ET_B_ receptor, are involved in SAH-induced vasospasm. ABT-627 is reported to greatly improve neurological deficits and reduce infarct size in mice after ischemic stroke, suggesting that the drug could normalize the vasoconstriction effect of ET-1 [28], as shown in the present study. Although a few ET_A_ receptor antagonists have been shown to have adverse effects in the SAH patients, we cannot rule out the possibility that there are pharmacological differences among ET_A_ receptor antagonists as well as testing conducted under non-optimized conditions. Here we report another possible ET_A_ receptor antagonist candidate, ABT-627, which may be an alternative therapeutic drug for treating SAH-induced vasospasm.

ET-1 stimulates the ET_B_ receptor and increases NO production [73]. NO is suggested to reverse the effects of the ET-1-induced vasoconstriction, by negative feedback and reducing the level of ET-1 [73,74]. Diminished production of NO and increased release of ET-1, therefore, are suggested to be the vital consequences of vasospasm in SAH [75]. It is reported that endothelial nitric oxide synthase (eNOS) expression as well as NO production are impaired by the elevated ET-1 level through a PKC-dependent pathway [42]. Since nitric oxide induces vascular relaxation by inhibiting PKC activity, the reduced NO production after SAH, therefore, enhances the PKC-dependent vasoconstriction [76]. Also, elevated level of ET-1 activates ET_A_ receptor and decreases the vascular sensitivity to NO by a PKC-independent pathway [77]. Down-regulation of PKC-α was observed in both Ntg and GET-1 MCA after ET_A_ receptor antagonist ABT-627 treatment (Figure 5C), suggesting that astrocytic ET-1 induced vasospasm is also mediated through ET_A_ receptor and PKC. These data collectively provide a strong rationale to investigate ABT-627 as a therapeutic drug to treat SAH-induced vasospasm.

K^+^ channels are important in regulating the membrane potential of arterial smooth muscle. Large conductance Ca^2+^-activated K^+^ channel is one of the channels that is found in the arterial smooth muscle and contributes to the resting membrane potential [45]. Recently, the function and expression of ion channels, particularly the Ca^2+^-activated K^+^ channels, have been investigated in SAH-induced vasospasm. In the present study, the expression of Ca^2+^-activated K^+^ channels in MCA was largely reduced after SAH in both Ntg and GET-1 mice, which is in agreement with others findings that elevated PKC activity after SAH causes dysfunction in K^+^ channel activity and expression [78,79].

**Figure 6:**
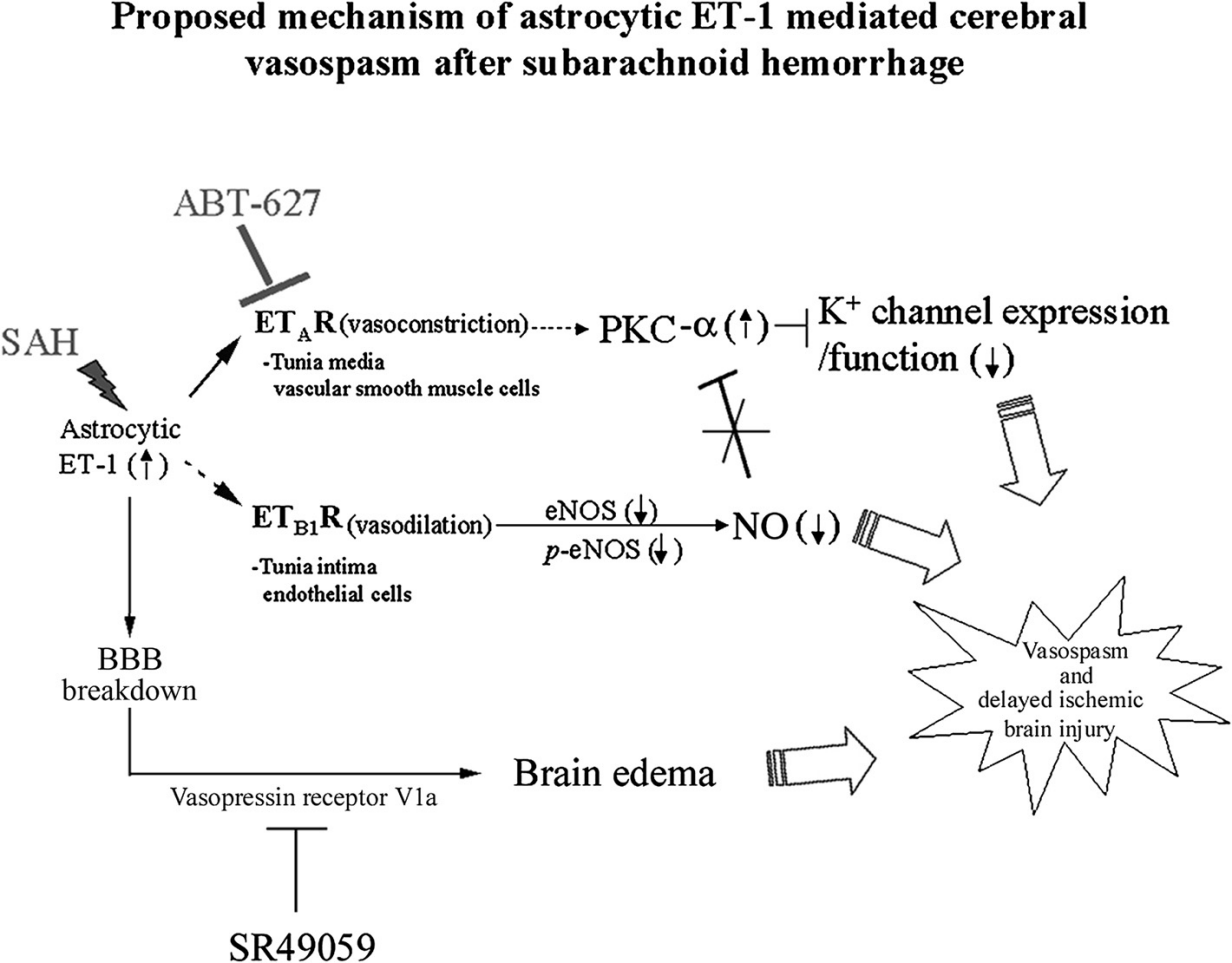
**Proposed mechanism of astrocytic ET-1 mediated cerebral vasospasm after SAH.** GET-1 mice with over-expressed in ET-1 showed more severe neurological deficits and vasospasm after SAH. Increased astrocytic ET-1 during SAH induces cerebral vasospasm through the ET_A_ receptor and mediated by PKC-α, which leads to dynfunction in the K^+^ channels. Administration of ET_A_ receptor antagonist ABT-627 ameliorates the SAH-induced vasospasm. The impairment of NO system also exaggerates the vasospasm effect. Astrocytic ET-1 leads to more severe cerebral edema and BBB breakdown that further contributes to cerebral vasoconstriction. Vasopressin V_1a_ receptor antagonist, SR 49059, significantly reduced the SAH-induced edema, suggesting that astrocytic ET-1 induces edema in SAH through vasopressin V_1a_ receptor.

Here we propose the possible mechanism of the astrocytic ET-1 mediated cerebral vasospasm after SAH (Figure 6). The astrocytic ET-1 induces cerebral vasospasm through the ET_A_ receptor, which leads to an upregulation of PKC-α.

## Conclusions

To the best of our knowledge, this is the first study to elucidate the role of astrocytic ET-1 in vasogenic edema formation and vasospasm development in SAH-induced brain injured mice. Both the vasopressin V_1a_ receptor antagonist, SR 49059, and ET_A_ receptor inhibitor, ABT-627 significantly ameliorated the astrocytic ET-1 induced complications after SAH. These potential therapeutic drugs could be used in future for treating SAH patients.

## Competing interests

The authors declare that they have no competing interests.

## Authors’ contributions

PKKY performed the majority of the experimental work, designed experiments, analysed data and wrote the manuscript. JS, SSMC and SKC conceived the idea, designed experiments and edited the manuscript. All authors read and approved the final manuscript.
